# Hybrid QuEChERS–Micro-Solid Phase Extraction for the Determination of Chloramphenicol in Honey by HPLC-MS/MS: Method Validation and Matrix-Effect Assessment

**DOI:** 10.3390/molecules31213971

**Published:** 2026-11-04

**Authors:** Tomasz Śniegocki, Elżbieta Samorek, Weronika Krajewska

**Affiliations:** Department of Chemical Research of Food and Feed, National Veterinary Research Institute, 24-100 Pulawy, Poland; elzbieta.samorek@piwet.pulawy.pl (E.S.); weronika.krajewska@piwet.pulawy.pl (W.K.)

**Keywords:** chloramphenicol, honey, QuEChERS, micro-solid phase extraction, HPLC-MS/MS, matrix effects

## Abstract

An HPLC-MS/MS method combining modified QuEChERS-based extraction with micro-solid-phase extraction (MSPE) using C18P-PVDF microspin filters was developed and validated for chloramphenicol (CAP) determination in honey. At validation levels of 0.10, 0.15, and 0.30 µg kg^−1^, apparent recoveries ranged from 102.1% to 105.1%, repeatability (RSDr) from 4.5% to 9.5%, and within-laboratory reproducibility (RSDwR) from 6.0% to 12.5%. The decision limit (CCα) was 0.123 µg kg^−1^, below the reference point for action of 0.15 µg kg^−1^, while the estimated limit of detection (LOD; not experimentally verified) was 0.01 µg kg^−1^.^.^ Matrix-effect assessment across 20 honey samples showed responses within the 80–120% range for the proposed procedure and the SPE and QuEChERS comparators. No CAP was detected in the authentic honey samples. The developed workflow provides a practical, validated alternative for routine CAP determination in honey.

## 1. Introduction

Chloramphenicol (CAP) is a broad-spectrum antibiotic whose use in food-producing animals is prohibited in the European Union due to its toxicological profile, including the risk of aplastic anaemia [1]. The presence of CAP isomers can complicate its selective identification [2,3]. Consequently, sensitive and selective confirmatory methods are required for monitoring CAP residues at or below the reference point for action (RPA) of 0.15 µg kg^−1^, as defined in the current European regulatory framework [4,5]. Honey is a challenging analytical matrix due to its high sugar content, pigments, and various minor constituents that may interfere with chromatographic separation and mass spectrometric detection [6,7]. These components can cause ion suppression or enhancement, affecting analytical response and quantification by HPLC-MS/MS [8].

The QuEChERS (Quick, Easy, Cheap, Effective, Rugged, and Safe) approach is widely used as a practical alternative to liquid–liquid extraction (LLE) and solid-phase extraction (SPE), offering relatively simple sample preparation and satisfactory analytical performance [9,10]. However, co-extracted matrix components may remain after QuEChERS-based preparation, particularly in complex matrices such as honey [8,11]. Several clean-up strategies have been investigated to reduce matrix interferences [12,13,14]. In the present study, a modified QuEChERS-based extraction was combined with micro-solid-phase extraction (MSPE) using C18P-PVDF microspin filters. The aim was to develop, optimise, and validate this procedure for the determination of CAP in honey by HPLC-MS/MS, with particular emphasis on method performance at and below the RPA. Matrix effects were compared with those obtained using SPE and QuEChERS procedures in the same 20 honey samples. Six additional dark honey samples were also examined to extend the matrix-effect assessment.

## 2. Results and Discussion

### 2.1. Validation Results

Repeatability, within-laboratory reproducibility, trueness (apparent recovery), and measurement uncertainty were evaluated at three concentration levels (0.10, 0.15, and 0.30 µg kg^−1^), below, at, and above the RPA of 0.15 µg kg^−1^, and are summarised in Table 1. The coefficient of determination (R^2^) for the matrix-matched calibration curve was 0.998, indicating satisfactory linearity within the tested working range. Repeatability (RSDr) ranged from 4.5% to 9.5%, while within-laboratory reproducibility (RSDwR) ranged from 6.0% to 12.5%. Apparent recoveries ranged from 102.1% to 105.1%, confirming satisfactory trueness of the method.

Ruggedness was assessed at 0.15 µg kg^−1^ using an eight-run Youden-type design involving deliberate variations in six sample preparation parameters. The measured CAP concentrations ranged from 0.135 to 0.156 µg kg^−1^, corresponding to CAP-D5-corrected apparent recoveries of 90.0–104.0%. The mean apparent recovery was 96.4%, with an RSD of 4.8%, indicating satisfactory method ruggedness under the investigated conditions. The largest estimated factor effects were associated with the methanol content of the loading solvent (−7.8 percentage points) and the elution solvent (+3.2 percentage points). These effects represent differences in CAP-D5-corrected apparent recovery between the higher and lower factor levels, expressed in percentage points. Detailed results are provided in Supplementary Table S2.

The decision limit (CCα), calculated using Method 3 of Commission Implementing Regulation (EU) 2021/808 [4], was 0.123 µg kg^−1^, below the RPA. The expanded measurement uncertainty, calculated at all three validation levels using a coverage factor of 2, corresponding to a confidence level of approximately 95%, ranged from 0.02 to 0.05 µg kg^−1^ (Table 1). The limit of detection (LOD) was estimated at 0.01 µg kg^−1^ by extrapolation from signal-to-noise data obtained at the lowest validation level. This estimate was not experimentally verified at the estimated detection limit.

### 2.2. Comparison with Previously Developed Methods

Selected HPLC-MS/MS methods previously reported for chloramphenicol determination in honey are summarised in Table 2. These include classical SPE-based clean-up, solvent extraction, online sample pretreatment, salting-out assisted liquid–liquid extraction (SALLE), and QuEChERS-based procedures. One example is the SALLE method reported by Rizzo et al. [15]. The published methods generally provided satisfactory recovery or accuracy; however, matrix-effect data were not reported consistently across studies. The apparent recoveries obtained with the proposed QuEChERS–MSPE procedure (102.1–105.1%) were comparable to those reported for previously developed methods. Nevertheless, differences in the analysed samples and approaches used to assess matrix effects limit direct comparisons of clean-up performance between these procedures.

The published methods differ primarily in their sample preparation and clean-up strategies and the complexity of individual procedural steps. The proposed method combines modified QuEChERS-based extraction with an additional clean-up step using C18P-PVDF microspin filters. Unlike dispersive approaches, the sorbent remains within the device, allowing for sample loading, analyte retention, and elution through successive centrifugation steps. This workflow represents a modification of existing sample preparation strategies rather than a new extraction principle. Its applicability to chloramphenicol determination in honey was evaluated through method validation and direct comparison with SPE and QuEChERS procedures using the same set of 20 honey samples.

### 2.3. Selectivity and Matrix Effect

**Figure 1:**
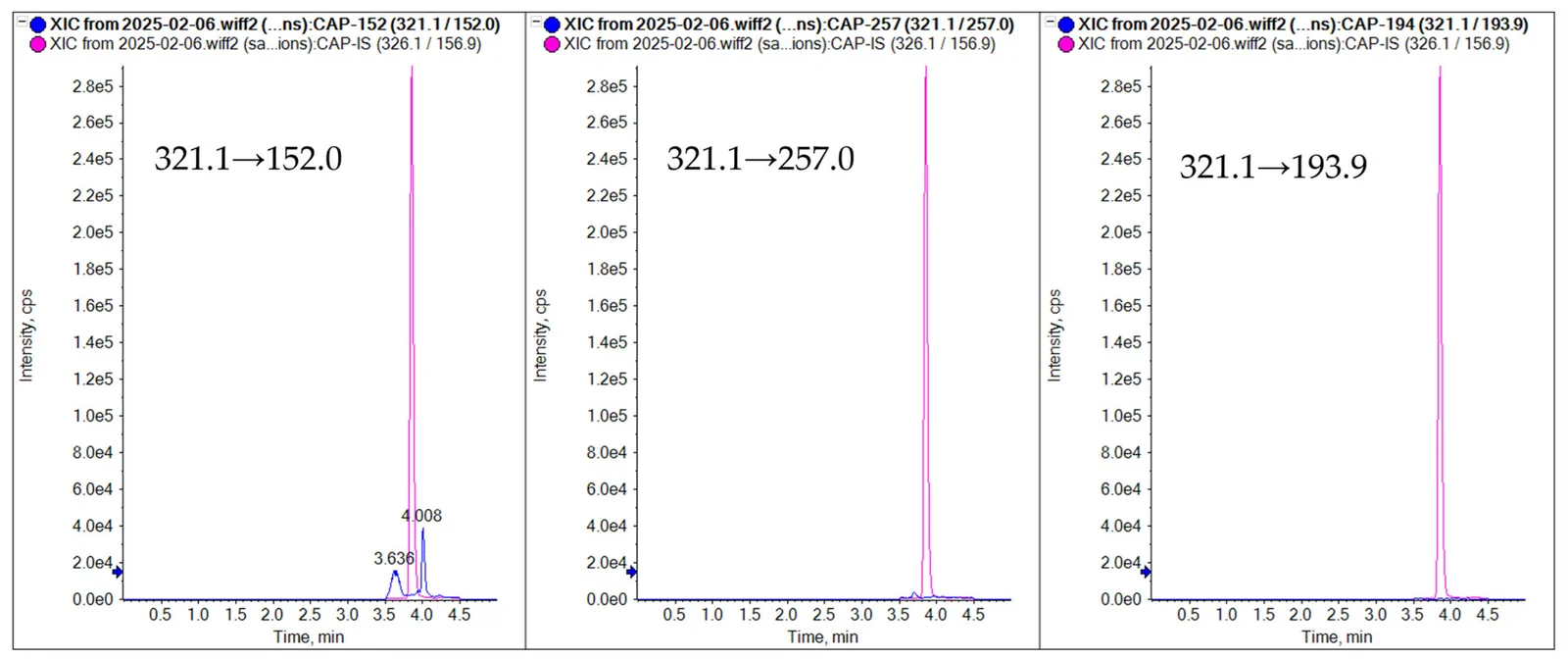
MRM chromatograms of a blank honey sample containing the internal standard CAP-D5 without added CAP. The CAP transitions *m*/*z* 321.1 → 152.0, 321.1 → 257.0, and 321.1 → 193.9 (**left** to **right**) are overlaid with the CAP-D5 transition (*m*/*z* 326.06 → 156.9).

**Figure 2:**
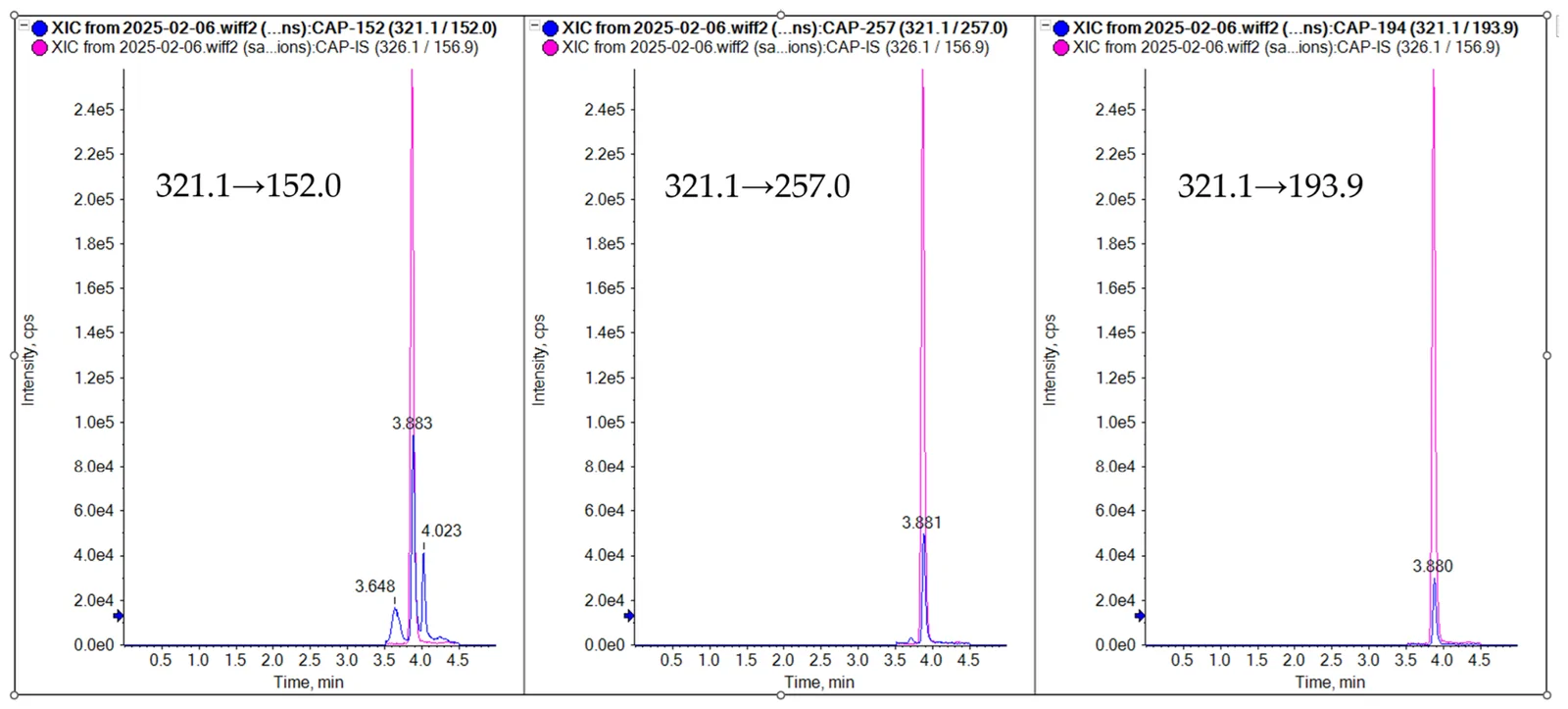
MRM chromatograms of a honey sample fortified with CAP at 0.15 µg kg^−1^ and containing the internal standard CAP-D5. The CAP transitions *m*/*z* 321.1 → 152.0, 321.1 → 257.0, and 321.1 → 193.9 (**left** to **right**) are overlaid with the CAP-D5 transition (*m*/*z* 326.06 → 156.9). CAP eluted at approximately 3.88 min.

Method selectivity was evaluated using 20 blank honey samples. In all 20 samples, additional signals were observed at approximately 3.64 and 4.02 min in the CAP quantifier transition (*m*/*z* 321.1 → 152.0). These signals were chromatographically separated from the CAP peak at approximately 3.88 min, with separation approaching the baseline. Automatic peak integration using SCIEX OS did not include the additional signals in the CAP peak area. No corresponding peaks were observed at matching retention times in the qualifier transitions (*m*/*z* 321.1 → 257.0 and 321.1 → 193.9), preventing the calculation of reliable qualifier-to-quantifier peak-area ratios. These signals therefore did not meet the identification criteria for CAP. Their chemical identities were not established. CAP-D5, which coeluted with CAP, was used as the retention-time reference for CAP identification. At all three validation levels, including 0.10 µg kg^−1^, relative retention-time deviations remained within ±1%, relative ion-intensity deviations were within ±40% of the reference ratios, and the signal-to-noise ratio was at least 10 for each diagnostic MRM transition. Representative chromatograms of a blank sample and a sample fortified with CAP at 0.15 µg kg^−1^ are shown in Figure 1 and Figure 2, respectively. Supplementary Figure S1 provides chromatograms of the same blank honeydew honey sample prepared using SPE, QuEChERS, and QuEChERS–MSPE. The honeydew honey sample was purchased from a retail store. A signal at approximately 3.92 min in the *m*/*z* 321.1 → 257.0 transition was observed following QuEChERS preparation but was not evident in the corresponding SPE chromatogram. This signal did not meet the identification criteria for CAP and was therefore considered a chromatographic interference. Its chemical identity and exact origin remain unknown. The method was also applied to 20 authentic honey samples from Poland, including multifloral, acacia, and rapeseed honeys. CAP was not detected in any of these samples.

**Figure 3:**
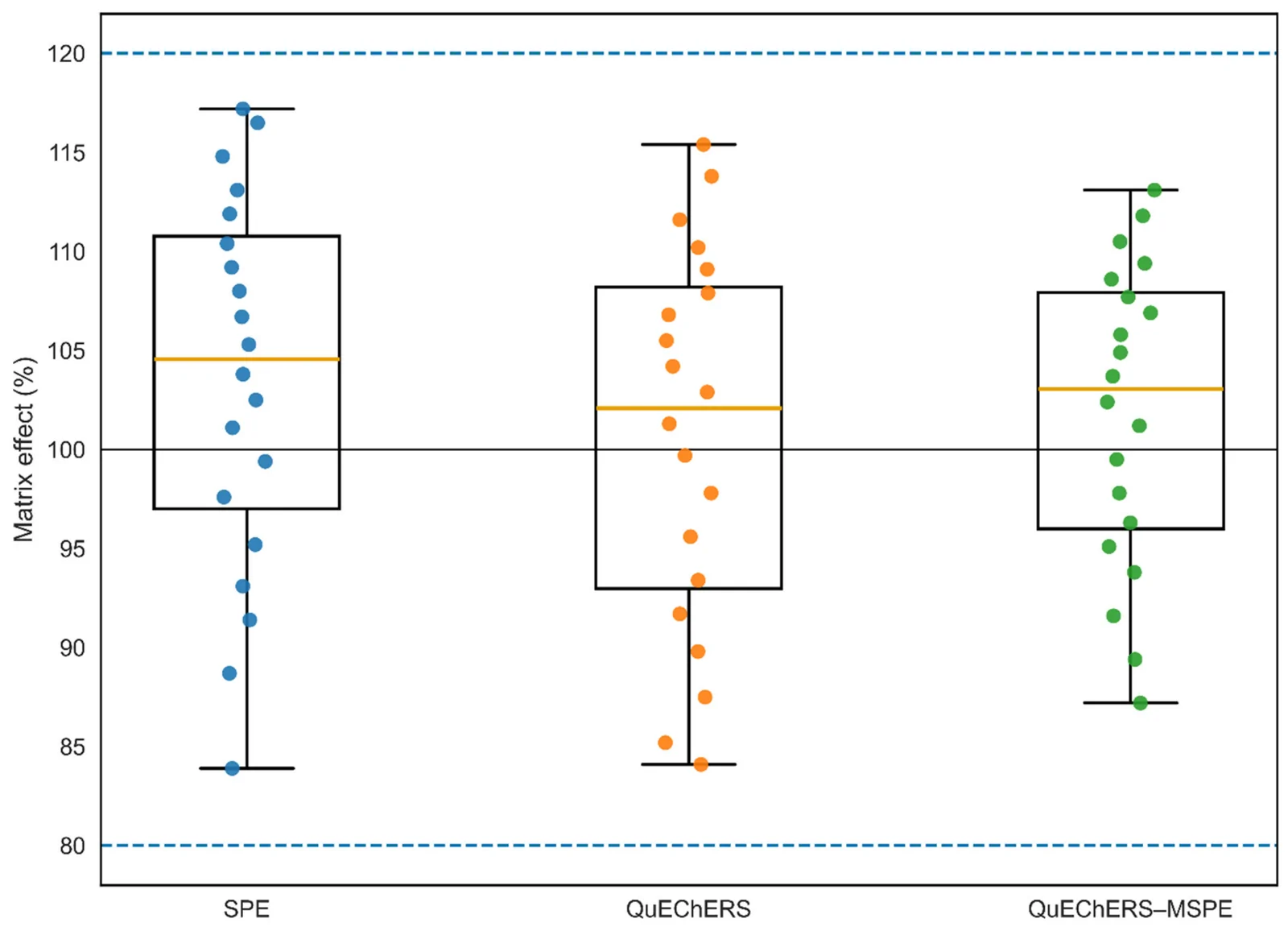
Distribution of CAP-D5-corrected matrix-effect values obtained for 20 honey samples prepared using SPE, QuEChERS, and QuEChERS–MSPE. The same set of samples was analysed using all three procedures. Individual data points are overlaid on boxplots. Dashed lines indicate the 80–120% range.

The same 20 honey samples, representing multifloral, acacia, and rapeseed honeys, were used to evaluate matrix effects. Each sample was prepared using SPE, QuEChERS, and QuEChERS–MSPE, allowing for direct comparison of the three procedures. Matrix-effect values calculated from CAP/CAP-D5 peak area ratios remained within the 80–120% range for all samples and procedures (Figure 3). QuEChERS–MSPE showed the lowest between-sample variability (SD = 7.71 percentage points), compared with SPE (9.59) and QuEChERS (9.67). The mean absolute deviation from the nominal 100% response was also lowest for QuEChERS–MSPE (6.77 percentage points), compared with SPE (8.56) and QuEChERS (8.20). However, these differences were not statistically significant. The Pitman–Morgan test yielded *p* = 0.185 for SPE versus QuEChERS–MSPE and *p* = 0.199 for QuEChERS versus QuEChERS–MSPE. The corresponding paired Wilcoxon signed-rank tests gave *p* = 0.186 and *p* = 0.213, respectively.

The matrix-effect assessment was extended to six dark honey samples, including three buckwheat and three honeydew honeys. Extracts prepared using QuEChERS and QuEChERS–MSPE were fortified with CAP and CAP-D5 after sample preparation and compared with reference solutions at matched concentrations. The mean absolute matrix effect, calculated from CAP peak areas without internal-standard correction, was 76.88% for QuEChERS and 85.52% for QuEChERS–MSPE. After CAP-D5 correction, the corresponding values were 85.13% and 95.35%, respectively. Both approaches yielded values closer to 100% following QuEChERS–MSPE, indicating lower ion suppression in the investigated samples. However, the difference in absolute matrix effects was not statistically significant (paired *t*-test, *p* = 0.396). In contrast, CAP-D5-corrected matrix-effect values were significantly higher following QuEChERS–MSPE than QuEChERS in the same six samples (paired *t*-test, *p* = 0.008).

### 2.4. Discussion

Combining modified QuEChERS-based extraction with C18P-PVDF microspin clean-up provided satisfactory apparent recovery and precision for CAP determination in honey at concentrations relevant to residue monitoring. The estimated LOD of 0.01 µg kg^−1^ was of the same order of magnitude as the values of 0.02 µg kg^−1^ reported by Barreto et al. and Huang et al. for CAP determination in honey [22,23]. Compared with previously developed SPE and QuEChERS procedures [20,21], the additional MSPE step represents a modification of the extract clean-up approach. In the direct comparison using 20 honey samples, all three procedures yielded CAP-D5-corrected matrix-effect values within the 80–120% range. QuEChERS–MSPE showed the lowest between-sample variability, although the differences were not statistically significant. In the additional experiment involving six dark honey samples, matrix-effect values were closer to 100% following QuEChERS–MSPE than QuEChERS, both before and after CAP-D5 correction. The difference in absolute matrix effects was also not statistically significant. In contrast, the difference in CAP-D5-corrected matrix effects was statistically significant in these six samples (paired *t*-test, *p* = 0.008). Comparison of the responses before and after internal-standard correction further highlights the role of CAP-D5 in compensating for matrix-related effects during CAP determination [8]. Nevertheless, CAP-D5 correction did not completely compensate for matrix effects, as indicated by the corrected values deviating from 100% in individual honey samples.

Comparison of C18P microspin filters equipped with PVDF, PTFE, and nylon membranes showed similar apparent CAP recoveries (103.6–106.2%), whereas more pronounced differences were observed in result variability. PVDF showed the lowest RSD (2.5%), compared with PTFE (9.7%) and nylon (8.4%). With PVDF, both extract loading and elution could be performed under the same centrifugation conditions (268× *g*, 3 min). PTFE required a higher centrifugation speed during extract loading, whereas nylon required a higher speed during elution. PVDF was therefore selected based on its low result variability and the ability to perform both clean-up steps under the same centrifugation conditions (Supplementary Table S1).

An important consideration in chloramphenicol determination is its discrimination from isomers that may exhibit similar fragmentation patterns. Berendsen et al. [2] demonstrated that distinguishing individual CAP isomers requires appropriate chromatographic conditions, while the relevance of this issue to honey analysis was also discussed by Yanovych et al. [3]. In the present method, CAP identification was based on retention time and the relative intensities of the monitored MRM transitions. The chromatographic conditions were not evaluated for the separation of all CAP isomers.

The proposed procedure was optimised and validated for CAP determination. Preliminary experiments with thiamphenicol and florfenicol also yielded satisfactory apparent recoveries under the same MSPE conditions (30% methanol for sample loading and 70% methanol for elution). These findings suggest that the procedure may also be applicable to other amphenicols. Extension to other classes of veterinary drugs would require further optimisation, particularly of sorbent selection and loading and elution conditions, due to differences in the physicochemical properties of the analytes.

Method validation was performed using CAP-fortified honey samples, while its application to authentic samples included 20 multifloral, acacia, and rapeseed honeys. Additional experiments with six buckwheat and honeydew honey samples extended the matrix-effect assessment to two other honey types. However, the influence of geographical origin, storage conditions, and honey processing on method performance was not systematically investigated. CAP was not detected in any of the authentic samples, and the new procedure was not evaluated using naturally contaminated samples, certified reference materials, or proficiency-testing materials. Further studies involving a broader range of honey samples would allow for a more comprehensive assessment of its applicability to routine residue monitoring.

## 3. Materials and Methods

### 3.1. Reagents and Materials

Chloramphenicol (CAP) and chloramphenicol-d5 (CAP-D5) were obtained from Dr Ehrenstorfer (LGC Labor GmbH, Augsburg, Germany). Stock standard solutions were prepared in methanol (1 mg mL^−1^) and stored at temperatures below −18 °C for a maximum of 12 months. Working solutions of CAP (0.1 µg mL^−1^) and the internal standard CAP-D5 (0.01 µg mL^−1^) were prepared in methanol and stored at 2–8 °C for a maximum of 6 months. Acetonitrile and methanol (LC-MS grade), sodium chloride (NaCl; analytical grade), and other analytical-grade reagents were obtained from J. T. Baker (Avantor Performance Materials Poland S.A., Gliwice, Poland), as described previously [21]. Isopropanol was obtained from VWR Chemicals (Avantor Performance Materials Poland S.A., Gliwice, Poland), and acetic acid from Honeywell (Charlotte, NC, USA). C18P microspin filters (P denotes Polar; the C18 phase is additionally modified to improve interactions with polar compounds) equipped with PVDF membranes were supplied by Chromservis (Jakobiho, Prague, Czech Republic) and used for MSPE clean-up. Ultrapure water was obtained using a Milli-Q system (Merck, Burlington, MA, USA).

### 3.2. HPLC-MS/MS Conditions

The HPLC-MS/MS system consisted of an AB Sciex ExionLC AE system coupled to an AB Sciex API 6500+ QTRAP mass spectrometer (AB Sciex, Concord, ON, Canada). SCIEX OS software (version 3.4.0.19154) was used for instrument control and data processing. Chromatographic separation was performed on a Phenyl–Hexyl column (50 × 2.1 mm, 5 µm) equipped with a SecurityGuard ULTRA Phenyl guard cartridge (2.0 mm, for 2.1 mm ID columns), both from Phenomenex (Torrance, CA, USA). The mobile phases consisted of 0.1% acetic acid in water (A) and methanol (B). The initial composition was 20% B, maintained for 2.0 min, followed by a linear increase to 80% B at 4.0 min. The initial composition (20% B) was restored at 4.2 min and maintained until the end of the run at 7.0 min to allow for column re-equilibration. The column temperature was 40 °C, the flow rate was 0.4 mL min^−1^, and the injection volume was 5 µL. Detection was performed in multiple reaction monitoring (MRM) mode using the following transitions: CAP (*m*/*z* 321.1 → 152.0, 321.1 → 257.0, and 321.1 → 193.9) and CAP-D5 (*m*/*z* 326.06 → 156.9). Negative electrospray ionisation (ESI−) was used with the following source parameters: spray voltage, −4000 V; source temperature, 450 °C; curtain gas, 40 psi; and ion source gases 1 and 2, 50 psi each. The declustering potential (DP) was −40 V, and the entrance potential (EP) was −10 V. The collision energies (CEs) were −24 eV for CAP-152.0 and CAP-D5, −16 eV for CAP-257.0, and −17 eV for CAP-193.9.

### 3.3. Sample Preparation

After thorough mixing to ensure sample homogeneity, 2 g of honey was transferred into a centrifuge tube and spiked with 30 µL of the CAP-D5 internal standard working solution (0.01 µg mL^−1^). Subsequently, 1.5 mL of ultrapure water was added to hydrate the sample. Extraction was performed using a modified QuEChERS-based procedure with 10 mL of acetonitrile and 0.5 g of NaCl. The samples were vortex-mixed for 5 min and centrifuged at 2930× *g* for 10 min at 6 °C. An 8 mL aliquot of the supernatant was collected and evaporated under a nitrogen stream at 45 ± 5 °C. The residue was reconstituted in 0.35 mL of 30% methanol before MSPE clean-up.

### 3.4. MSPE Clean-Up Procedure

The C18P-PVDF microspin filter was conditioned with 0.7 mL of 30% methanol. The reconstituted extract (0.35 mL in 30% methanol) was then loaded onto the conditioned filter and centrifuged at 268× *g* for 3 min at room temperature. The flow-through fraction was discarded, and the collection tube was replaced. CAP was eluted with 0.2 mL of 70% methanol under the same centrifugation conditions (268× *g*, 3 min). No additional washing step was included. The evaluated conditions and selected settings are summarised in Table 3.

### 3.5. Method Validation

The method was validated according to Commission Implementing Regulation (EU) 2021/808 [4]. The evaluated parameters included selectivity, working range, repeatability, within-laboratory reproducibility, apparent recovery, ruggedness, decision limit (CCα) and measurement uncertainty. Selectivity was assessed using 20 blank honey samples from Poland, representing acacia, multifloral, and rapeseed honeys. No signals meeting the CAP identification criteria were detected at the expected retention time. No matrix-related interferences affecting CAP identification were observed in honey samples fortified at 0.15 µg kg^−1^. Confirmatory identification was based on retention time and the relative intensities of the monitored MRM transitions, in accordance with Regulation (EU) 2021/808. The relative retention time of CAP with respect to CAP-D5 was required to agree with that of the corresponding calibration or matrix-matched standard within ±1%. The relative intensities of the diagnostic MRM transitions were required to agree within ±40% relative deviation, with a minimum signal-to-noise ratio of 10 for each diagnostic transition.

The working range was evaluated using matrix-matched calibration curves prepared at six concentration levels: 0.10, 0.15, 0.30, 0.60, 1.20, and 2.00 µg kg^−1^. Calibration curves were freshly prepared for each validation series. The internal standard was added at the same level to calibration samples, fortified samples, and blanks before extraction. Each calibration level was analysed in seven independent replicates, and calibration curves were constructed using weighted (1/x) linear regression. Repeatability was evaluated at three concentration levels (0.10, 0.15, and 0.30 µg kg^−1^), with seven independently prepared samples analysed at each level within a single analytical run. Within-laboratory reproducibility was evaluated over three days by two operators. Seven independently fortified samples were analysed at each concentration level on each day, resulting in 21 determinations per validation level (63 determinations across the three levels). Apparent recovery was calculated as the percentage ratio of the measured CAP concentration to the corresponding fortified concentration.

Ruggedness was evaluated at a CAP concentration of 0.15 µg kg^−1^ using an eight-run Youden-type experimental design. Deliberate variations were introduced in six sample preparation parameters: methanol content of the loading solvent, methanol content of the elution solvent, elution volume, reconstitution volume, mixing time, and centrifugation conditions. The nominal values and investigated ranges were 30% (25–35%) methanol in the loading solvent, 70% (65–75%) methanol in the elution solvent, 0.20 mL (0.18–0.22 mL) elution volume, 0.35 mL (0.30–0.40 mL) reconstitution volume, 5 min (4–6 min) mixing time, and 268× *g* (approximately 217–325× *g*) centrifugation force. The calculated factor effects are provided in Supplementary Table S2. C18P microspin filters equipped with PVDF, PTFE, and nylon membranes were evaluated separately using eight independently prepared honey samples fortified at 0.15 µg kg^−1^ for each membrane. Measurements were performed in four analytical batches, with two independent preparations per batch. Centrifugation conditions were adjusted according to membrane requirements. Apparent recoveries were corrected using CAP-D5. The membrane comparison results are presented in Supplementary Table S1.

The decision limit (CCα) was determined according to Method 3 of Section 2.6 of Commission Implementing Regulation (EU) 2021/808 [4], using the equation CCα = LCL + 2.33 × uc(LCL), where LCL is the lowest calibration level (0.10 µg kg^−1^) and uc(LCL) is the combined standard uncertainty at that level. Measurement uncertainty was estimated at the three validation levels (0.10, 0.15, and 0.30 µg kg^−1^), considering type A and type B contributions, including within-laboratory precision, analytical reference standards, preparation of calibration solutions, volumetric measurements, sample weighing, and recovery-related bias. For routine analysis, results were reported as the mean of two independently prepared test portions. The uncertainty estimation accounted for the reduction in the relevant random components through averaging, while retaining common and systematic contributions. Expanded measurement uncertainty was calculated using a coverage factor of k = 2.

### 3.6. Matrix-Effect Assessment and Comparison of Sample Preparation Procedures

Matrix effects were evaluated in 20 honey samples by comparing SPE, QuEChERS, and QuEChERS–MSPE sample preparation procedures. The SPE and QuEChERS procedures were based on previously published methods [20,21]. Each sample was prepared using all three procedures, with the final extract solvent composition standardised across workflows and the extracts analysed within the same HPLC-MS/MS sequence. The internal-standard-corrected matrix effect was calculated as the CAP/CAP-D5 peak-area ratio in matrix-matched standards divided by the corresponding ratio in solvent-based standards, multiplied by 100% [8]. Values below and above 100% indicated negative and positive residual matrix effects after CAP-D5 correction, respectively.

An additional matrix-effect assessment was performed using six dark honey samples, including three buckwheat and three honeydew honeys. Each honey sample was processed using both QuEChERS and QuEChERS–MSPE, providing paired matrix-effect measurements. Blank extracts prepared using QuEChERS and QuEChERS–MSPE were fortified with CAP and CAP-D5 after sample preparation and compared with corresponding neat reference solutions matched for analyte concentrations and solvent composition. CAP and CAP-D5 were added at concentrations corresponding to 0.15 µg kg^−1^ in the original honey sample. The absolute matrix effect was calculated as the CAP peak area in the matrix extract divided by the CAP peak area in the neat reference solution, multiplied by 100%. For absolute matrix effects, values below 100% indicated ion suppression, whereas values above 100% indicated signal enhancement. The CAP-D5-corrected matrix effect was also determined for the same samples using CAP/CAP-D5 peak-area ratios.

For the comparison involving 20 honey samples, between-sample matrix-effect variability was characterised by the standard deviation, while deviations from the nominal 100% response were assessed using the mean absolute deviation. Differences in variability between procedures were evaluated using the Pitman–Morgan test. Absolute deviations from 100% were compared using the paired Wilcoxon signed-rank test with exact *p*-values. For the six dark honey samples, differences in both absolute and CAP-D5-corrected matrix effects between QuEChERS and QuEChERS–MSPE were evaluated using a paired *t*-test.

## 4. Conclusions

Combining modified QuEChERS-based extraction with C18P-PVDF microspin clean-up provided a procedure for the determination of chloramphenicol in honey. The method showed satisfactory apparent recovery and precision at the investigated concentration levels, with a decision limit (CCα) of 0.123 µg kg^−1^, below the reference point for action (RPA) of 0.15 µg kg^−1^. In the direct comparison of three sample preparation procedures, QuEChERS–MSPE showed the lowest variability in CAP-D5-corrected matrix effects, although the differences were not statistically significant. In the six dark honey samples, QuEChERS–MSPE showed lower ion suppression than QuEChERS, although the difference was not statistically significant. In contrast, CAP-D5-corrected matrix-effect values were significantly higher following QuEChERS–MSPE. These findings support C18P-PVDF microspin clean-up as an alternative sample preparation approach for CAP determination in honey. Further evaluation is needed to assess its applicability to other analytes and a broader range of honey types.

## Figures and Tables

**Table 1:** Validation results for determination of chloramphenicol in honey at three fortification levels.

Parameter	Fortification Level (µg kg^−1^)		
	0.10	0.15	0.30
Repeatability (RSDr, %)	9.5	6.8	4.5
Within-laboratory reproducibility (RSDwR, %)	12.5	9.8	6.0
Apparent recovery (%)	104.3 ± 2.4	102.1 ± 3.5	105.1 ± 3.1
Expanded uncertainty (µg kg^−1^, k = 2)	0.02	0.03	0.05
CCα (µg kg^−1^)	0.123	–	–

**Table 2:** Comparison of the proposed method with previously developed approaches for chloramphenicol determination.

Ref.	Clean-Up	Recovery/Accuracy (%)	Matrix-Effect Data	Main Feature
[16]	SPE + LLE	Accuracy within ±15%	NR	Multistep SPE
[17]	C18 SPE	98.7–102.0	Ion suppression 60–70%	High recovery
[18]	Hexane + ACN	91–104	NR	Rapid workflow
[19]	Online MC-ODS	Recovery ~78%; accuracy 92–111	NR	Online clean-up
[20]	SPE	92.1–107.1	8.56 *	Robust clean-up
[21]	QuEChERS	93.1–108.0	8.20 *	Multi-matrix
[15]	SALLE	>90	NR	Low-cost extraction
This work	QuEChERS–MSPE	102.1–105.1	6.77 *	C18P-PVDF microspin clean-up

* Values marked with an asterisk represent the mean absolute deviation of CAP-D5-corrected matrix-effect values from 100%, expressed in percentage points. These values were obtained in the present study using the same 20 honey samples for SPE, QuEChERS, and QuEChERS–MSPE. Recovery/accuracy data for previously published methods and unmarked matrix-effect data were taken from the corresponding cited studies. NR, not reported.

**Table 3:** Optimisation of sample preparation and MSPE conditions for chloramphenicol determination in honey.

Parameter	Conditions Evaluated	Selected Condition
Membrane material	PVDF, PTFE, nylon	PVDF
Loading solvent	20, 25, 30, 35 and 40% MeOH	30% MeOH
Elution solvent	50, 55, 60, 65, 70, 75 and 80% MeOH	70% MeOH
Elution volume	0.15, 0.20, 0.25, 0.30 and 0.35 mL	0.20 mL
Reconstitution volume	0.20, 0.25, 0.30, 0.35, 0.40, 0.45 and 0.50 mL	0.35 mL
Mixing time	2, 5 and 10 min	5 min
Centrifugation	151, 268, 604, 1358× *g* (3 min)	268× *g* (3 min)

## Data Availability

The data presented in this study are available from the corresponding author upon reasonable request.

## Supplementary Materials

The following supporting information can be downloaded at https://www.mdpi.com/article/10.3390/molecules31213971/s1. Figure S1: Full MRM chromatograms of the same blank honeydew honey sample prepared using (A) SPE, (B) QuEChERS–MSPE, and (C) QuEChERS. Columns show CAP transitions *m*/*z* 321.1 → 152.0, 321.1 → 257.0, and 321.1 → 193.9 (left to right), each overlaid with CAP-D5 (*m*/*z* 326.06 → 156.9). Additional peaks in the first transition have no corresponding signals in the other CAP transitions at matching retention times. Table S1: Comparison of C18P microspin filters equipped with different membranes for chloramphenicol analysis in honey. Table S2: Eight-run ruggedness assessment of six MSPE parameters for chloramphenicol determination in honey.
