# Supplementary material for: Hybrid QuEChERS–Micro-Solid Phase Extraction for the Determination of Chloramphenicol in Honey by HPLC-MS/MS: Method Validation and Matrix-Effect Assessment
Source: Molecules. 2026 Nov 4;31(21):3971. doi: 10.3390/molecules31213971 (PMC13764750; doi:10.3390/molecules31213971)
Supplement: Supplementary file 1 [file molecules-31-03971-s001.zip › molecules-4624601-supplementary.pdf]

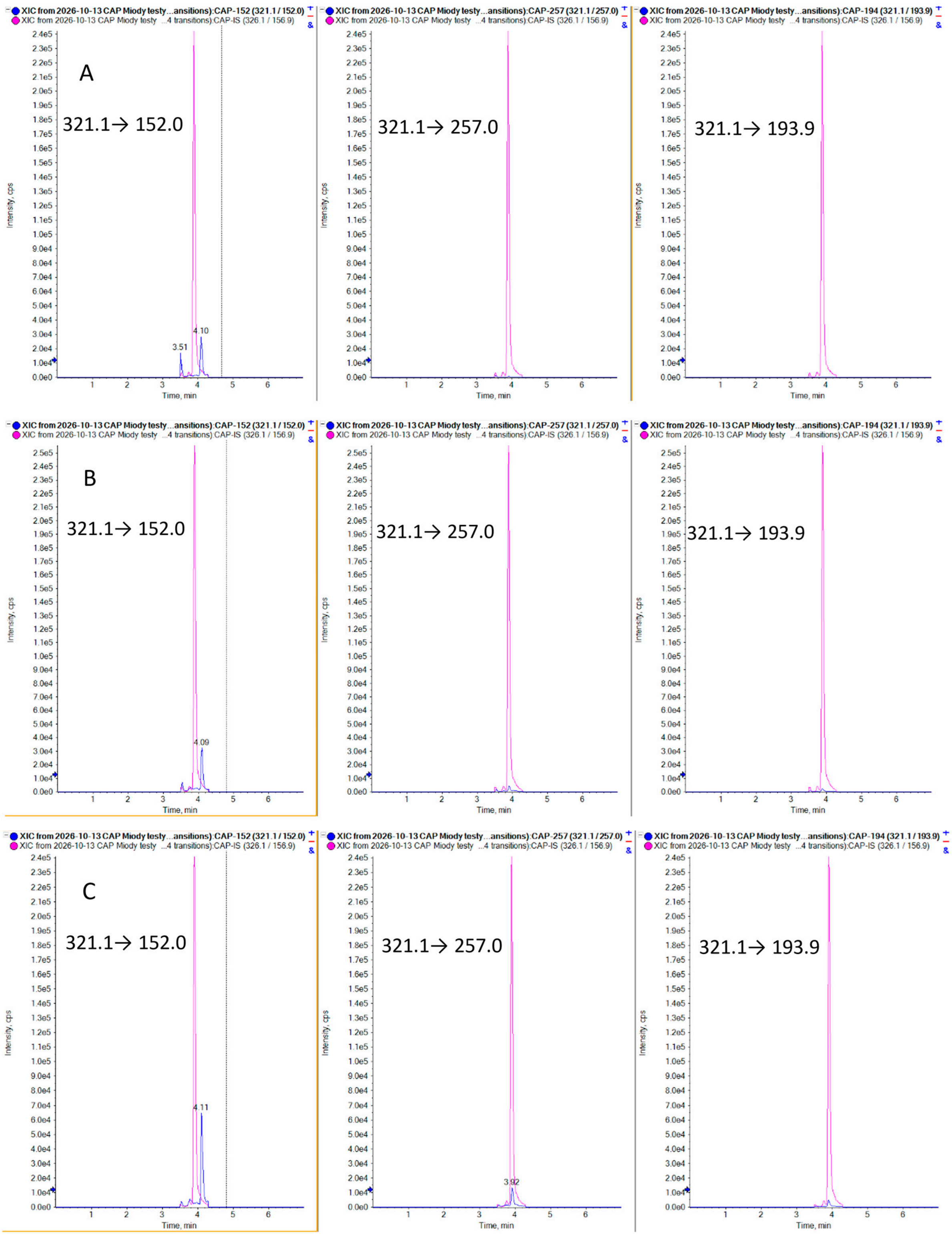

Figure S1. Full MRM chromatograms of the same blank honeydew honey sample prepared using (A) SPE, (B) QuEChERS–MSPE, and (C) QuEChERS. Columns show CAP transitions m/z 321.1 → 152.0, 321.1 → 257.0, and 321.1 → 193.9 (left to right), each overlaid with CAP-D5 (m/z 326.06 → 156.9). Additional peaks in the first transition have no corresponding signals in the other CAP transitions at matching retention times.

Table S1. Comparison of C18P microspin filters equipped with different membranes for chloramphenicol analysis in honey.

**Membrane**	**Independent preparations (n)**	**Mean apparent recovery (%)**	**RSD (%)**
PVDF	8	103.6	2.5
PTFE	8	104.5	9.7
Nylon	8	106.2	8.4

Note. Samples were fortified at 0.15 μg kg^{-1}. Apparent recoveries were corrected using CAP-D5. Measurements comprised four analytical batches with two independently prepared test portions in each batch (n = 8 per membrane). Centrifugation conditions were adjusted when required for extract transfer; therefore, the comparison concerns the evaluated membrane workflows, not membrane material under strictly identical centrifugation conditions.

Table S2. Eight-run ruggedness assessment of six MSPE parameters for chloramphenicol determination in honey.

Factor	Nominal	Lower level	Higher level	Signed effect (pp)
MeOH in loading solvent (%)	30	25	35	−7.8
MeOH in elution solvent (%)	70	65	75	+3.2
Elution volume (mL)	0.20	0.18	0.22	−0.9
Reconstitution volume (mL)	0.35	0.30	0.40	+0.2
Mixing time (min)	5	4	6	−0.1
Centrifugation (× g)	268	217	325	+0.3

Note. Eight experiments were conducted at a CAP fortification level of 0.15 μg kg^{-1}. Overall CAP-D5-corrected apparent recovery was 96.4% (range 90.0–104.0%; RSD 4.8%). Factor effects are reported as signed changes in apparent recovery in percentage points (pp), following the ruggedness analysis. Centrifugation forces for the varied levels are approximate conversions for the same rotor, using the nominal relation 2000 rpm = 268 × g.
